# Association Between Stress‐Induced Weight Loss and Autophagy‐Related Gene Expression in the Hippocampus and Midbrain of Depression Model Mice

**DOI:** 10.1002/npr2.12515

**Published:** 2024-12-23

**Authors:** Hiroaki Mori, Yuta Yoshino, Mariko Okano, Yu Funahashi, Hiroshi Kumon, Shinichiro Ochi, Jun‐ichi Iga, Shu‐ichi Ueno

**Affiliations:** ^1^ Department of Neuropsychiatry, Molecules and Function Ehime University Graduate School of Medicine Toon Japan

**Keywords:** autophagy, chronic immobilization stress (CIS), depression, Fkbp5, stress‐induced weight loss

## Abstract

**Aim:**

Recent studies have implicated autophagy in both weight regulation and depression. This study aimed to investigate the relationship between stress‐induced weight loss and autophagy‐related gene expression in a mouse model of depression.

**Method:**

Male C57BL/6 mice were subjected to a chronic immobilization stress (CIS) protocol for 14 days to induce depressive‐like behavior. Body weight was measured before and after the CIS, and depressive‐like behavior was assessed using the tail suspension test (TST). The expression levels of autophagy‐related genes (*Atg5*, *Atg7*, *Atg12*, *Becn1*, *Mmp9*, *Fkbp5*, and *Map1lc3b*) in the hippocampus and midbrain were evaluated using reverse transcription‐quantitative PCR (RT‐qPCR). Serum cortisol levels were also measured.

**Results:**

The CIS resulted in significant weight loss and increased immobility time in the TST, indicating depressive‐like behavior. Serum cortisol levels were not different between CIS‐depression model and control mice. In the hippocampus, the expression levels of *Fkbp5*, *Mmp9*, and *Map1lc3b* were significantly higher in CIS‐depression model mice than in control mice. In the midbrain, the expression levels of *Fkbp5* and *Mmp9* were significantly higher in CIS‐depression model mice than in control mice. Increased autophagy‐related gene expressions in CIS‐depression model mice were consistent with the previous studies in the postmortem brains of patients with depression. A significant negative correlation was also found between *Fkbp5* mRNA expression in the hippocampus and the weight change ratio before and after the CIS.

**Conclusion:**

The findings suggest that enhanced autophagy may be related to the pathology of depression and that Fkbp5, an autophagy regulator, mediates stress‐induced weight loss.

## Abstract 2

This study examined the effects of chronic immobilization stress (CIS) on mouse behavior, weight, and autophagy‐related gene expression in the hippocampus. In the CIS response group, a correlation analysis was conducted between weight change ratio and Fkbp5 mRNA expression levels, using Pearson's correlation coefficient. Results showed a significant correlation, with significance defined at a *p* value< 0.05.

## Graphical abstract

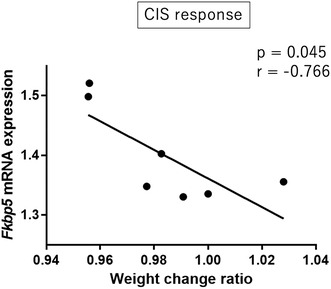

## 1 Introduction

Major depressive disorder (MDD) is a mental disease that has become the major cause of disability worldwide [1, 2]. Symptoms of MDD often include loss of appetite and weight loss [3, 4], and these are also observed in some animal models [5]. However, the relationship between body weight and MDD is complex and multifaceted. While rodents often overeat due to acute psychological stress [6], chronic psychological stress reduces food intake, body weight, and adiposity in rodents [7, 8]. Melancholic depression is characterized by intense loss of appetite and weight, whereas atypical depression is the opposite, characterized by increased appetite and weight [9]. A systematic review and meta‐analysis found that both underweight and obesity increase the risk of MDD [10]. Another study indicated that fat mass is a risk factor for MDD [11].

In recent years, autophagy has been the focus of much attention in studies of both body weight and MDD [12, 13, 14]. Autophagy is a vital intracellular pathway for cellular homeostasis through the lysosomal degradation of damaged macromolecules and organelles [15]. Neuronal cells are particularly vulnerable to autophagy deficiency [16]. Thus, autophagy has been linked to several psychiatric diseases including MDD [12, 16]. Previous studies showed increased expression of autophagy‐related genes in mononuclear cells in patients with MDD [17]. In animal models, chronic mild unpredictable stress in mice has been reported to enhance hippocampal autophagy [18]. Antidepressants have been reported to induce autophagy, suggesting that autophagy may play an important role in the pathology and treatment of MDD [19, 20]. The pharmacological inhibition of Beclin1, an autophagy marker, abrogated the beneficial cellular and behavioral effects of antidepressants in mice [21].

In addition, it has been shown that autophagy is related to body weight regulation and eating behavior [13, 14, 22]. Fasting or calorie restriction can cause adaptive autophagy and prolong the longevity of eukaryotic cells [22]. Dysfunctional autophagy in the hypothalamus has been linked to obesity and related metabolic complications [13]. Deletion of FK‐506 binding protein 51 (Fkbp51), the regulator protein of autophagy, in the mouse hypothalamus was a strong inducer of obesity, whereas its overexpression prevented high‐fat diet‐induced obesity [23]. Autophagy promotes an organism's resilience to weight gain caused by high‐calorie diets [24].

Although weight loss is a well‐known phenomenon as a symptom of psychological distress and MDD, its mechanism has not been clarified. This study aimed to determine how stress‐induced weight loss altered autophagy in the hippocampus and midbrain of depression model mice. In this study, mice underwent a chronic immobilization stress (CIS) protocol for 14 days. Mice whose immobilization time was significantly longer in the TST than in the control group were considered depression model mice. Weight changes before and after the CIS protocol were compared between control and depression model mice. The expression of autophagy‐related genes (*Atg5*, *Atg7*, *Atg12*, *Becn1*, *Mmp9*, *Fkbp5*, and *Map1lc3b*), which are reported to be upregulated in the brains of MDD patients [25, 26, 27], were examined in the hippocampus and midbrain of control and depression model mice. Furthermore, the correlations of the gene expressions with TST immobilization time and body weight change were investigated.

## 2 Materials and Methods

### 2.1 Animal Care

Male C57BL/6 mice (8 weeks old) were purchased from CLEA Japan (Tokyo, Japan). The mice were housed in a pathogen‐free environment with a 12‐h light/12‐h dark cycle, room temperature (22°C), 50% humidity, and climate control. The animals were allowed to habituate to the room for 1 week before experimental manipulations were performed. The mice were provided with water and a standard diet (Oriental Yeast Co. Ltd., Tokyo, Japan).

### 2.2 Experimental Procedure for Chronic Immobilization Stress (CIS)

The mice were subjected to the CIS protocol (*n* = 12) or a home‐cage control protocol (*n* = 9) for 14 days (Day 1–Day 14). The CIS protocol was based on previous studies [28]. Briefly, the mice in the CIS groups were subjected to immobilization stress by restraint using a plastic cylinder with a diameter of 3 cm and a height of 12 cm. The animals were subjected to 6 h of immobilization stress (8:30–14:30) once daily for 14 days (Day 1–Day 14). The control group mice were also deprived of water and food during the 6 h of the CIS protocol.

### 2.3 Body Weight Measurement and Tail Suspension Test (TST)

Body weight measurements were performed for all mice before the CIS protocol (Day 0) and after the CIS protocol (Day 14). The weight change ratio was determined using the following equation: Weight change ratio = the weight of the mouse on day 14/the weight of the mouse on day 0. All mice underwent TST after the CIS protocol (Day 14). The TST was conducted between 19:00 and 24:00. Each mouse was individually suspended from a fixed hook using a small piece of adhesive tape placed approximately 2 cm from the tip of the tail. The immobility of the mice was assessed with an automated system (Bioseb, Vitrolles, France) [29] during the 4 min between 2 and 6 min after starting the session. The mice assigned to the CIS protocol with longer than the average immobilization time of the control group mice were defined as “CIS response mice,” who were also regarded as depression model mice. The mice assigned to the CIS protocol with shorter than the average immobilization time in the control group were removed from further analysis. On Day 16, the mice were anesthetized and killed by decapitation. The Animal Experiment Committee of Ehime University approved all the animal experiments (Approval number: 05TU231).

### 2.4 Plasma Cortisol Measurement

We measured the plasma cortisol of mice to evaluate the effects on the HPA axis by CIS. Although it is well‐known that the primary glucocorticoid in rodents is corticosterone [30], we chose plasma cortisol because a previous study suggested that corticosterone declines gradually during repeated restraints, whereas cortisol remains at a high level [31]. On Day 16, a blood sample was collected from the heart of each mouse in an EDTA tube after decapitation. Collected blood samples were centrifuged, and plasma was collected and stored at −80°C. Plasma cortisol levels were quantitatively analyzed with a RayBio mouse cortisol EIA kit (RayBiotech, Peachtree Corners, GA, USA).

### 2.5 RNA Isolation, Synthesis of Complementary DNA (cDNA)

After decapitation, each hippocampus and midbrain was dissected and immediately frozen at −80°C for RNA isolation. RNA was isolated according to the previously reported protocol [32]. TRIzol (Thermo Fisher Scientific, Waltham, MA, USA) was used for RNA isolation. RNA concentration and quality were calculated using the NanoDrop 1000 system (Thermo Fisher Scientific). Then, cDNA was synthesized using the High‐Capacity cDNA Reverse Transcription Kit (Applied Biosystems, Foster City, CA, USA). To synthesize cDNA, RNA (1.0 μg) was used in 40‐μL reaction mixtures.

### 2.6 Reverse Transcription‐Quantitative PCR (RT‐qPCR)

The mRNA expressions of genes associated with autophagy (*Atg5, Atg7, Atg12, Becn1, Mmp9, Fkbp5*, and *Map1lc3b*) were examined by RT‐qPCR using the StepOnePlus Real‐Time PCR System (Applied Biosystems). These autophagy‐related genes were selected based on reports that their expressions were increased in the brains of depressed patients [25, 26, 27]. The Predesigned qPCR assay (Integrated DNA Technologies, Coralville, IA, USA) used Mm.PT.58.45861921 for *Fkbp5*, Mm.PT.58.10100097 for *Mmp9*, Mm.PT.33504448 for *Becn1*, Mm.PT.58.32352062 for *Atg7*, Mm.PT.58.31606064 for *Atg12*, Mm.PT.58.8305340 for *Atg5*, Mm.PT.58.29764292 for *Map1lc3b*, and Mm.PT.39a.1 for *Gapdh*. RT‐qPCR was conducted using the PrimeTime Gene Expression Master Mix (Integrated DNA Technologies) in a final volume of 10 μL. The mRNA expression levels were measured in duplicate. To remove errors between plates, the same sample was included as a calibrator in each plate. The ΔΔCt method [33] was used to calculate the relative expression value.

### 2.7 Statistical Analysis

The SPSS 29.0 software (IBM Japan, Tokyo, Japan) was used for statistical analysis. The normality of the distribution was evaluated using the Shapiro–Wilk test. Student's *t*‐test or the Mann–Whitney *U* test was used to compare the average mRNA expression levels between CIS response and control mice. The correlation of *Fkbp5* mRNA expression with the weight change ratio was evaluated using Pearson's correlation analysis. A significance threshold of *p* value < 0.05 was used.

## 3 Results

### 3.1 CIS Induced Depressive‐Like Behavior and Weight Loss

**FIGURE 1:**
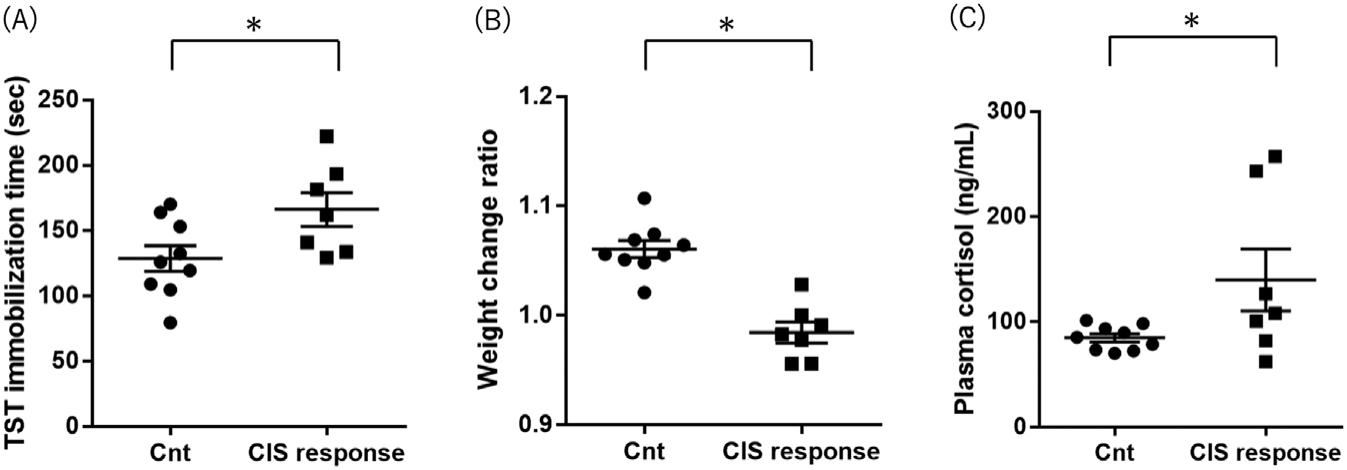
The effects of CIS on behavior, weight, and plasma cortisol levels. (A) Comparison of TST immobility time between the control and CIS response groups. The y‐axis shows TST immobility time in seconds. (B) Comparison of the weight change ratio between the control and CIS response groups. The y‐axis shows the relative weight change ratio (=the weight of the mouse on day 14/the weight of the mouse on day 0). (C) Comparison of plasma cortisol levels between the control and CIS response groups. The y‐axis shows the plasma cortisol level in ng/mL. Significance was defined at *p* value < 0.05. The horizontal lines and error bars represent the means ± standard error. CIS, chronic immobilization stress; Cnt, control; TST, tail suspension test (*p value < 0.05, Student's *t*‐test).

CIS response mice (*n* = 7) with longer than the average immobilization time in the control group mice were regarded as depression model mice. The data of TST immobility time and weight are shown in Figure 1 and Table S1. TST immobility time differed significantly between the control and CIS response mice (Student's *t*‐test *p* value < 0.001; Figure 1A and Table S1). Although the weight of mice before the CIS protocol was not different between control and CIS response group mice (Student's *t*‐test *p* value = 0.304, Table S1), the weight after the CIS protocol was significantly lower in the CIS response group mice than in the control group mice (Mann–Whitney *U* test *p* value < 0.001, Table S1). The weight change ratio in the CIS response group was downregulated compared with the control group (Student's *t*‐test *p* value < 0.001; Figure 1B and Table S1). There was no significant difference in plasma cortisol levels between the CIS response group and the control group (Student's *t*‐test *p* value = 0.111; Figure 1C and Table S1).

### 3.2 CIS Changed Autophagy‐Related Gene Expressions in the Hippocampus

**FIGURE 2:**
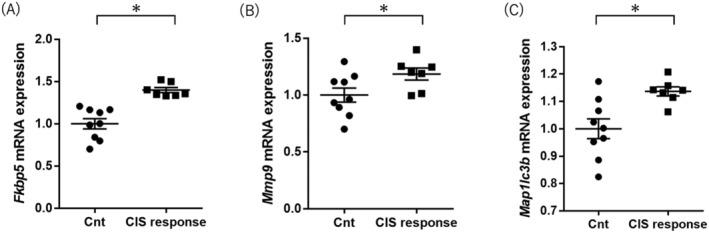
Comparison of (A) *Fkbp5*, (B) *Mmp9*, and (C) *Map1lc3b* mRNA expressions in the hippocampus between the control and CIS response groups. The y‐axis shows the relative expression level (/the mean of Cnt mice) from the qPCR results. Significance was defined at *p* value < 0.05. The horizontal bars represent the means ± standard error. CIS, chronic immobilization stress; Cnt, control (*p value < 0.05, Student's *t*‐test).

*Fkbp5* expression levels were higher in the CIS response mice than in the control group mice (Student's *t*‐test *p* value < 0.001; Figure 2A and Table 1). *Mmp9* expression levels were higher in CIS response mice than in control group mice (Student's *t*‐test *p* value = 0.048; Figure 2B and Table 1). *Map1lc3b* expression levels were significantly higher in CIS response mice than in control group mice (Student's *t*‐test, *p* value = 0.008; Figure 2C and Table 1). These increased expression gene changes (*Fkbp5*, *Mmp9*, and *Map1lc3b*) in the CIS response group were consistent with the results in postmortem brains of MDD patients [25, 26, 27]. *Atg5*, *Atg7*, Atg12, and *Becn1* expression levels were not different between the two groups (Table 1).

**TABLE 1:** Expression levels of autophagy‐related genes in hippocampus.

mRNA expression	Control (*n* = 9)	CIS response (*n* = 7)	*p*
*Fkbp5*	1.00 ± 0.18	1.40 ± 0.08	< 0.001*
*Mmp9*	1.00 ± 0.19	1.22 ± 0.19	0.048*
*Atg5*	1.00 ± 0.06	1.05 ± 0.06	0.133
*Atg7*	1.00 ± 0.07	1.06 ± 0.05	0.114
*Atg12*	1.00 ± 0.05	0.97 ± 0.07	0.288
*Becn1*	1.00 ± 0.10	1.03 ± 0.06	0.511
*Map1lc3b*	1.00 ± 0.11	1.14 ± 0.04	0.008*

*Note:* Values are means ± standard deviation.

Abbreviation: CIS, chronic immobilization stress.

^*^ *p* value < 0.05, Student's *t*‐test or Mann–Whitney *U* test.

### 3.3 CIS Changed Autophagy‐Related Gene Expressions in the Midbrain

**FIGURE 3:**
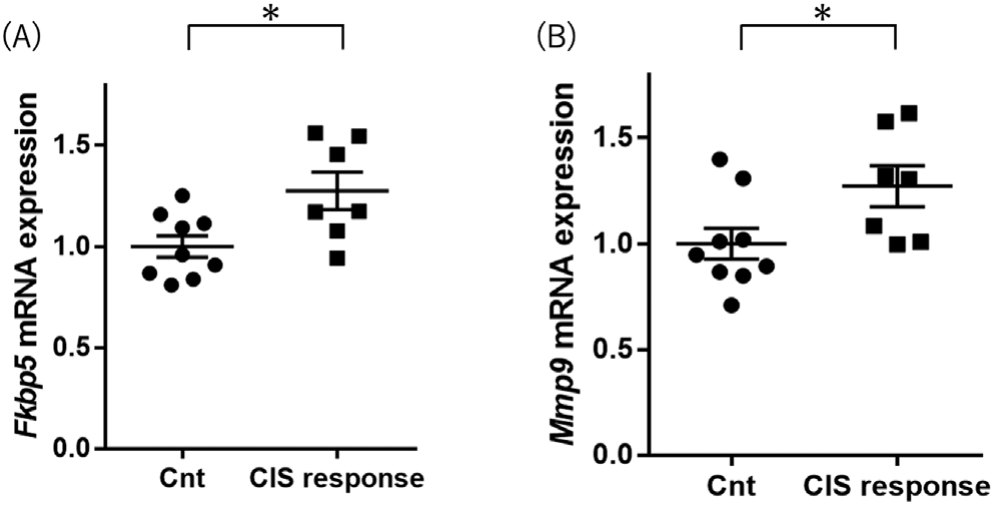
Comparison of (A) *Fkbp5* and (B) *Mmp9* mRNA expressions in the midbrain between the control and CIS response groups. The y‐axis shows the relative expression level (/the mean of Cnt mice) from the qPCR results. Significance was defined at *p* value < 0.05. The horizontal bars represent the means ± standard error. CIS, chronic immobilization stress; Cnt, control (*p value < 0.05, Student's *t*‐test).

*Fkbp5* expression levels were higher in the CIS response mice than in the control group mice (Student's *t*‐test *p* value = 0.016; Figure 3A and Table 2). *Mmp9* expression levels in CIS response mice were higher than in control group mice (Student's *t*‐test *p* value = 0.039; Figure 3B and Table 2). These increased expression gene changes (*Fkbp5* and *Mmp9*) in the CIS response group were consistent with the results in the hippocampus and the results in the postmortem brains of MDD patients [25, 27]. *Map1lc3b*, *Atg5*, *Atg7*, Atg12, and *Becn1* expression levels were not different between the two groups (Table 2).

**TABLE 2:** Expression levels of autophagy‐related genes in midbrain.

mRNA expression	Control (*n* = 9)	CIS response (*n* = 7)	*p*
*Fkbp5*	1.00 ± 0.16	1.28 ± 0.24	0.016*
*Mmp9*	1.00 ± 0.22	1.27 ± 0.26	0.039*
*Atg5*	1.00 ± 0.08	0.96 ± 0.05	0.321
*Atg7*	1.00 ± 0.07	0.99 ± 0.08	0.725
*Atg12*	1.00 ± 0.07	0.93 ± 0.09	0.111
*Becn1*	1.00 ± 0.07	1.00 ± 0.07	0.904
*Map1lc3b*	1.00 ± 0.06	1.05 ± 0.06	0.108

*Note:* Values are means ± standard deviation.

Abbreviation: CIS, chronic immobilization stress.

^*^ *p* value < 0.05, Student's *t*‐test.

### 3.4 Negative Correlation Between Fkbp5 mRNA Expression and the Weight Change Ratio

**FIGURE 4:**
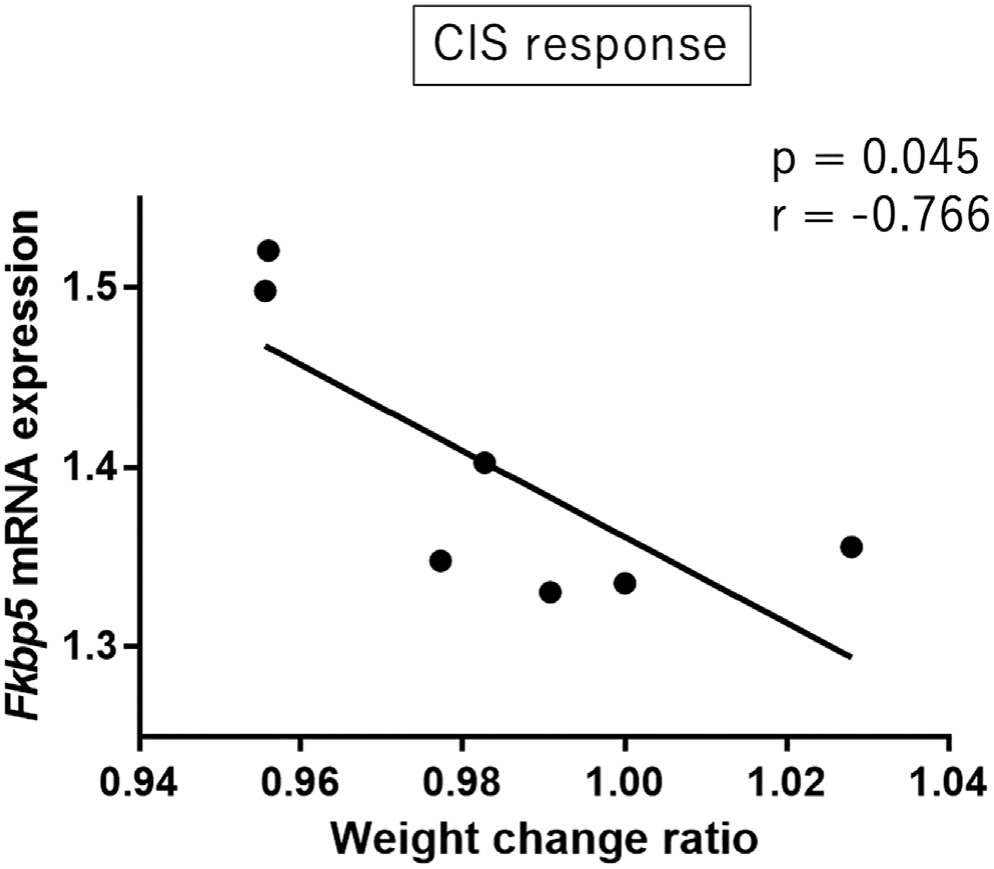
Correlation between the weight change ratio and *Fkbp5* mRNA expression of the hippocampus in CIS response group mice. The correlation was analyzed by Pearson's correlation coefficient. The x‐axis shows the weight change ratio (=the weight of the mouse on day 14/the weight of the mouse on day 0). The y‐axis is the relative expression level (/the mean of Cnt mice) from the qPCR results. Significance was defined at *p* value < 0.05. CIS, chronic immobilization stress.

The weight change ratio was significantly negatively correlated with Fkbp5 mRNA expression in the hippocampus of CIS response group mice (Pearson's correlation analysis: *r* = −0.766, *p* = 0.045; Figure 4). TST immobility time and Fkbp5 mRNA expression were not significantly correlated in CIS response group mice (Pearson's correlation analysis: *r* = 0.054, *p* = 0.908). Other autophagy‐related gene expressions in the hippocampus did not show any significant correlations with the weight change ratio or TST immobility time. In the midbrain, none of the autophagy‐related gene expressions showed significant correlations with the weight change ratio or TST immobility time.

## 4 Discussion

In recent years, autophagy has been highlighted in the study of MDD [12, 16]. There are substantially consistent results that autophagy markers are increased in MDD patients [17, 26]. Autophagy is a beneficial process of the stress response, but under certain conditions, excessive and chronic activation can be harmful [34, 35]. For example, exaggerated autophagy causes type II cell death [36]. Short‐term caloric restriction, one of the most effective triggers of autophagy, has been reported to have antidepressant effects in humans and mice [37, 38], whereas the effects of long‐term caloric restriction are detrimental [38]. It has been suggested that acute stress activates lytic autophagy, which has an initial beneficial effect by increasing neuroplasticity and cognitive alertness, while prolonged or chronic stress may induce neuroinflammation [35]. In addition, a previous report suggested that excessive autophagy reduces BDNF expression in neurons and disrupts hippocampal neurogenesis in corticosterone‐induced depression model mice [39].

Fkbp51, which is encoded by the *Fkbp5* gene, negatively regulates the HPA axis by inhibiting the interaction between GR and glucocorticoid hormone [40]. A previous study suggested that the inhibition of Fkbp51 by SAFit2, the selective antagonist of FKBP51, promotes stress resilience to chronic psychological stress in mouse models and might be a novel antidepressant [41]. Fkbp51 is also known to be an important upregulator protein of autophagy [42, 43]. It has been shown that upregulation of FKBP51 mediated glucocorticoid‐induced autophagy [42]. FKBP51 induces autophagy and exacerbates damage in cerebral ischemic stroke [43]. A previous study suggested that the deletion of Fkbp51 in the mouse hypothalamus strongly induced obesity by decreasing autophagy, whereas its overexpression prevented obesity by increasing autophagy [23].

Microtubule‐associated protein 1A/1B light chain 3B (Map1lc3b) is a major protein in the autophagy pathway and is most widely known as a marker of autophagosomes [44]. A previous report suggested that *Map1lc3b* mRNA expression in the brain was higher in MDD patients than in healthy controls [26]. Another report found that *Map1lc3b* mRNA expression was increased in the peripheral blood mononuclear cells of patients with MDD [17].

Matrix metallopeptidase 9 (Mmp9) protein is a matrixin involved in the degradation of the extracellular matrix in normal physiological processes [45] and is essential for autophagy [46]. A recent study showed that Mmp9 protein was enhanced by stress‐induced secretory autophagy [35]. It has also been thought that *Mmp9* might also be involved in the pathogenesis of MDD [25, 47, 48]. A previous study has shown that *Mmp9* mRNA expression and protein levels were upregulated in the blood of MDD patients [47]. Another study reported that increased MMP9 enzymatic activity was observed in the hippocampus of postmortem brain samples from MDD patients [48]. Mmp9 protein levels were increased in the postmortem brains of MDD patients treated with antidepressants compared with controls [25].

In the present study, *Fkbp5*, *MMP9*, and *Map1lc3b* mRNA expressions were upregulated in the depression model mice compared with the control mice. These results were consistent with the results seen in postmortem brains of MDD patients [25, 26, 27]. These results also coincide with the hypothesis that chronic and excess autophagy activation is harmful and leads to psychiatric disease [34, 35, 36, 39]. Although it is well‐studied that antidepressants also increase autophagy, it is thought that starting a process of neuronal reorganization that ultimately constitutes the transition from disease to health [49]. These differences in the effects of autophagy may be due to the duration and pathway [35, 49], but the mechanism remains unclear.

On the other hand, although the gene expressions of initial autophagy signaling (*Atg5*, *Atg7*, Becn1, and *Atg12*) in CIS mice were not significantly upregulated in the present study, most of these genes showed a tendency to increase compared to control mice. These could be explained by type II errors due to the small sample size or could indicate an abnormality in the autophagy pathway of depression and therefore require further analysis.

In the present study, *Fkbp5* mRNA expression was correlated with stress‐stimulated weight loss in depression model mice. Considering that *Fkbp5* is a regulator of body weight via autophagy [23], this result suggests that the increase in *Fkbp5* mRNA expression due to chronic psychological stress may cause weight loss through the promotion of autophagy. As the central brain region for metabolic and weight control, the hypothalamus has been well‐studied [13, 23, 50, 51]. On the other hand, the hippocampus and midbrain are well‐known as the central region for MDD [18, 52, 53, 54] and are also thought to be involved in weight and metabolic control nowadays [55, 56, 57]. Therefore, we chose the hippocampus and midbrain as the research region for the connection between weight loss and depression. Several studies have implicated the autophagy pathway in the hypothalamus involved in weight regulation [14, 23, 50] and it has been suggested that the dysregulation of autophagy in the hypothalamus may influence body weight through impaired leptin signaling [58], glucose intolerance [59], and impaired sympathetic control of the peripheral metabolic organs [60]. It is possible that autophagy in the hippocampus and midbrain affects body weight by mechanisms similar to those in the hypothalamus. To elucidate these mechanisms, further research is needed.

In our present study, there was no significant difference in plasma cortisol levels between the CIS response group and the control group. Based on the previous study [31], we assumed that plasma cortisol would remain at a high level during CIS, but the actual result was different. This result could be attributed to the short duration of CIS in our study. In the previous study [31], the plasma cortisol levels decreased once after 2 weeks of CIS and increased again after 3 weeks of CIS. There may have been a significant difference in plasma cortisol levels between the CIS response group and the control group if 3 weeks of CIS had been performed. The CIS protocols are often conducted for 2–4 weeks [61]. In this study, we chose the shortest duration of 2 weeks due to ethical considerations for the mice. Future studies will need to consider the duration of CIS.

The present study has some limitations. First, the small sample size may lead to type II errors. Second, it is difficult to determine the function of autophagy by autophagy‐related gene mRNA expressions, since assessing autophagic flux or turnover of long‐lived proteins is complicated in mice [44, 49]. Third, although *Fkbp5* mRNA expression was correlated with stress‐stimulated weight loss in depression model mice, it does not allow us to postulate a direct causal relationship between *Fkbp5* and weight loss because decreased appetite and food intake may be confounding factors. Due to technical limitations, the present study could not assess changes in food intake, body temperature, and activity of the mice. Further studies are needed to clarify the correlation between autophagy and stress‐induced weight loss.

## 5 Conclusions

The results of the present study show that the expressions of autophagy‐related genes (*Fkbp5, Mmp9, and Map1lc3b*) were increased in the hippocampus of a mouse model of depression that exhibited weight loss. The increased gene expression in the mouse model of depression was consistent with that observed in the postmortem brains of MDD patients. In particular, *Fkbp5* mRNA expression was correlated with stress‐induced weight loss in the depression model mice. These results suggest that enhanced autophagy may be related to the pathogenesis of MDD and that *Fkbp5* mediates stress‐induced weight loss.

## Author Contributions

Conceptualization: H.M. and J.‐i.I. Methodology: H.M., Y.Y., and J.‐i.I. Investigation: H.M., M.O., Y.F., H.K., Y.Y., and S.O. Writing – original draft preparation: H.M. Writing – review and editing: Y.Y., S.‐i.U., J.‐i.I., and H.M. Supervision: S.‐i.U. Funding acquisition: S.‐i.U. and J.‐i.I. All the authors contributed to and approved the final manuscript.

## Ethics Statement

Approval of the research protocol by an Institutional Reviewer Board: All animal experiments were approved by the Animal Experiment Committee of Ehime University (Approval number; 05TU231) and were performed under the Guidelines for Animal Experiments at Ehime University.

Animal Studies: This study complies with to Act on Welfare and Management of Animals and Ministry of Education, Culture, Sports, Science and Technology (MEXT)'s Fundamental Guidelines for Proper Conduct of Animal Experiment and Related Activities in Academic Research Institutions.

## Consent

The authors have nothing to report.

## Conflicts of Interest

The authors declare no conflicts of interest.

## Supporting information


Data S1.

Table S1.

## Data Availability

The data that supports the findings of this study are available in the supplementary material of this article.
