# Supplementary material for: Association Between Stress‐Induced Weight Loss and Autophagy‐Related Gene Expression in the Hippocampus and Midbrain of Depression Model Mice
Source: Neuropsychopharmacol Rep. 2024 Dec 23;45(1):e12515. doi: 10.1002/npr2.12515 (PMC11666342; doi:10.1002/npr2.12515)
Supplement: Supplementary file 2 — Table S1. [file NPR2-45-e12515-s002.docx]

**Supplementary Table 1.** TST immobility time, weight, and serum cortisol levels

|  | **Control**  **(n=9)** | **CIS response**  **(n=7)** | **p-value** |
| --- | --- | --- | --- |
| TST immobility time (s) | 128.6 ± 29.7 | 166.1 ± 34.6 | < 0.001* |
| Weight before CIS protocol (g) | 22.71± 0.95 | 22.3 ± 0.60 | 0.304 |
| Weight after CIS protocol (g) | 24.1 ± 0.87 | 21.9 ± 0.50 | < 0.001* |
| Weight change ratio | 1.06 ± 0.02 | 0.98 ± 0.02 | < 0.001* |
| Serum cortisol (ng/mL) | 84.8 ± 11.7 | 140.0 ± 78.0 | 0.111 |

Values are means ± standard deviation. Abbreviations: CIS, chronic immobilization stress; TST, tail suspension test. (*p-value < 0.05, Student’s *t*-test or Mann-Whitney U test).
